# Tri-Allelic Human Leukocyte Antigen (HLA) Protection Against Dementia

**DOI:** 10.29245/2572.942x/2019/1.1261

**Published:** 2019-12-27

**Authors:** Lisa M. James, Apostolos P. Georgopoulos

**Affiliations:** 1 Brain Sciences Center, Department of Veterans Affairs Health Care System, Minneapolis, USA; 2 Department of Neuroscience, University of Minnesota Medical School, Minneapolis, USA; 3 Department of Psychiatry, University of Minnesota Medical School, Minneapolis, USA; 4 Department of Neurology, University of Minnesota Medical School, Minneapolis, USA

**Keywords:** Human Leukocyte Antigen, HLA, Dementia, Persistent Antigens, Europe

## Abstract

Human Leukocyte Antigen (HLA) Class II DRB1*13:02 has recently been found to protect against dementia in Continental Western Europe. Here we extend those findings by evaluating the association between the population frequency of two additional Class II HLA alleles – DRB1*01:01 and DRB1*15:01 – alone and in combination with DRB1*13:02, on dementia prevalence in Continental Western Europe. Results indicated that the prevalence of dementia in 14 Continental Western European (CWE) countries significantly decreased exponentially with increasing frequency of any of the three alleles alone and in combination (P’s < 0.001). When combined, the population frequency of the three alleles accounted for 67% of the variance in dementia prevalence. The combined frequency of DRB1*01:01, DRB1*13:02, and DRB1*15:01 was also significantly associated with dementia prevalence in those aged 65 years and older (P = 0.004) and with a change in dementia prevalence between 1990 and 2016 (P = 0.006). These findings, which document the protective effects of three common Class II HLA alleles on dementia prevalence in CWE, are discussed in terms of the role of HLA class II genes in pathogen elimination. More specifically, we hypothesize that dementia prevalence is higher for countries in which the population frequency of these protective alleles is low, prohibiting the successful elimination of pathogens that may play a causal role in dementia.

## Introduction

Recent evidence points to immunogenetic protection against age-related brain changes^1,2^ and dementia^3^ conferred by human leukocyte antigen (HLA) DRB1*13:02, a class II allele. HLA genes, which are located in the Major Histocompatibility Complex (MHC) of chromosome 6, code for cell-surface glycoproteins that facilitate the elimination of foreign antigens and, therefore, play a critical role in host protection. Class II HLA alleles (DRB, DQB, DPB) present exogenous antigenic peptides, such as those derived from viruses and bacteria, to CD4 receptors to promote antibody production. Antibody production and subsequent elimination of foreign antigens, however, are predicated on good binding affinity between HLA and epitopes of foreign antigens coupled with the resulting immunogenicity. Sufficient affinity/immunogenicity results in immunological memory and host protection in the event of re-exposure; however, poor affinity/immunogenicity may lead to antigen persistence and, consequently, neuronal damage^4,5^ and possibly dementia^4,6^. Indeed, several microbes have been associated with Alzheimer’s dementia^7,8^ and pathogens have been implicated in other forms of dementia as well^6^. We have hypothesized that the observed DRB1*13:02 protection against age-related brain changes and dementia is conferred by successful elimination of foreign antigens and have speculated that other HLA genes may similarly protect against pathogens that may be associated with dementia.

The HLA region is the most highly polymorphic in the human genome, having evolved to maximize species resistance to foreign antigens^9,10^. Still, some alleles are considerably more common than others^11,12^, presumably due to evolutionary advantage in conferring host protection. DRB1*13:02, for example, is one of the most common DRB alleles and has been shown to be highly protective against several conditions including various autoimmune disorders^13,14^, Gulf War Illness^15,16^, and infectious diseases^17–19^. Furthermore, DRB1*13:02 has also been shown to protect against age-related brain atrophy^1^ and functional deterioration^2^ in cognitively healthy women, even after accounting for presence of apolipoprotein e4 (apoE4), a well-established genetic risk factor for dementia^20^. This evidence of protective effects against age-related brain changes led us to hypothesize that DRB1*13:02 may protect against dementia. Indeed, in a recent genetic epidemiological study we evaluated the relationship between the population frequency of DRB1*13:02 and dementia prevalence in 14 CWE countries and found that dementia prevalence decreased exponentially with increasing frequency of DRB1*13:02, even after adjusting for the prevalence of apoE4.^3^ Remarkably, the population frequency of DRB1*13:02 accounted for 45% of the variance in dementia prevalence. In the 14 countries included in that study^3^, the frequency of DRB1*13:02 ranged from 0.025–0.062, which equates to 5–13% carriers in the respective country population, suggesting that other factors, such as additional protective HLA alleles, may account for the remaining variance in dementia prevalence. Thus, in the present study, we evaluated the association between other common DRB1alleles and dementia prevalence in the same 14 CWE countries.

## Methods

### CWE countries.

As in our previous study^3^, we focused on the following 14 CWE countries: Austria, Belgium, Denmark, Finland, France, Germany, Greece, Italy, Netherlands, Portugal, Norway, Spain, Sweden, and Switzerland.

### Prevalence of dementia.

As with our prior study^3^, dementia prevalence was computed by dividing the number of people with dementia in 2016 in each country^21^ by the total population of the country in 2016^22^ and expressed as a percentage. In the present study, we also estimated dementia prevalence for those aged 65 and older by adjusting the total population prevalence obtained above by the percent of the population that is 65 or older^22^. These data are shown in Table 1. We have previously shown that life expectancy for these countries are virtually identical^3^ (Table 1); therefore, life expectancy was not included in the current analyses.

### ApoE4 prevalence.

The apoE4 prevalence was obtained from published data^23^ and is shown in Table 2.

### HLA alleles.

In our previous study^3^ we identified DRB1*13:02 as having a protective role on dementia prevalence in the 14 CWE counties above. Here we sought to find out additional HLA DRB1 alleles, if any, with such a protective effect on dementia prevalence. For that purpose, we searched the publicly available database of immune gene frequencies (Allele Frequency Net Database, http://www.allelefrequencies.net) for DRB1 alleles that were genotyped at high (4 digit) resolution and were available for all the 14 CWE countries above. This search (on October 19, 2019) yielded 6 alleles (DRB1*01:01, DRB1*10:01, DRB1*11:01, DRB1*13:01, DRB1*13:02, DRB1*14:01) with complete data. We also found 5 alleles for which data were available for 13 countries (DRB1*03:01, DRB1*04:01, DRB1*08:01, DRB1*12:01, DRB1*15:01); specifically, data for DRB1*03:01, DRB1*04:01, DRB1*08:01, and DRB1*12:01 were missing for Switzerland, and data for DRB1*15:01 were missing for Belgium. We could not find any data in the web for the Swiss-missing alleles but we did find DRB1*15:01 frequency for Belgium from a research publication^24^. Thus we evaluated the relation to dementia prevalence for the following 7 alleles: DRB1*01:01, DRB1*10:01, DRB1*11:01, DRB1*13:01, DRB1*13:02, DRB1*14:01, and DRB1*15:01. Of those 7 alleles, only three (DRB1*01:01, DRB1*13:02 and DRB1*15:01) had a statistically significant protective effect on dementia prevalence (see below) and were analyzed further. The frequencies of these alleles for each country are given in Table 3.

### Statistical analyses.

Regression and correlation analyses were used to evaluate the association between the population frequency of each of the three HLA alleles, DRB1*01:01, DRB1*13:02, and DRB1*15:01 (alone and combined), and the prevalence of dementia in Continental Western Europe. All statistical analyses were conducted using the IBM-SPSS statistical package (version 23).

## Results

### Unique HLA allele effects on dementia prevalence

**Figure 1.**
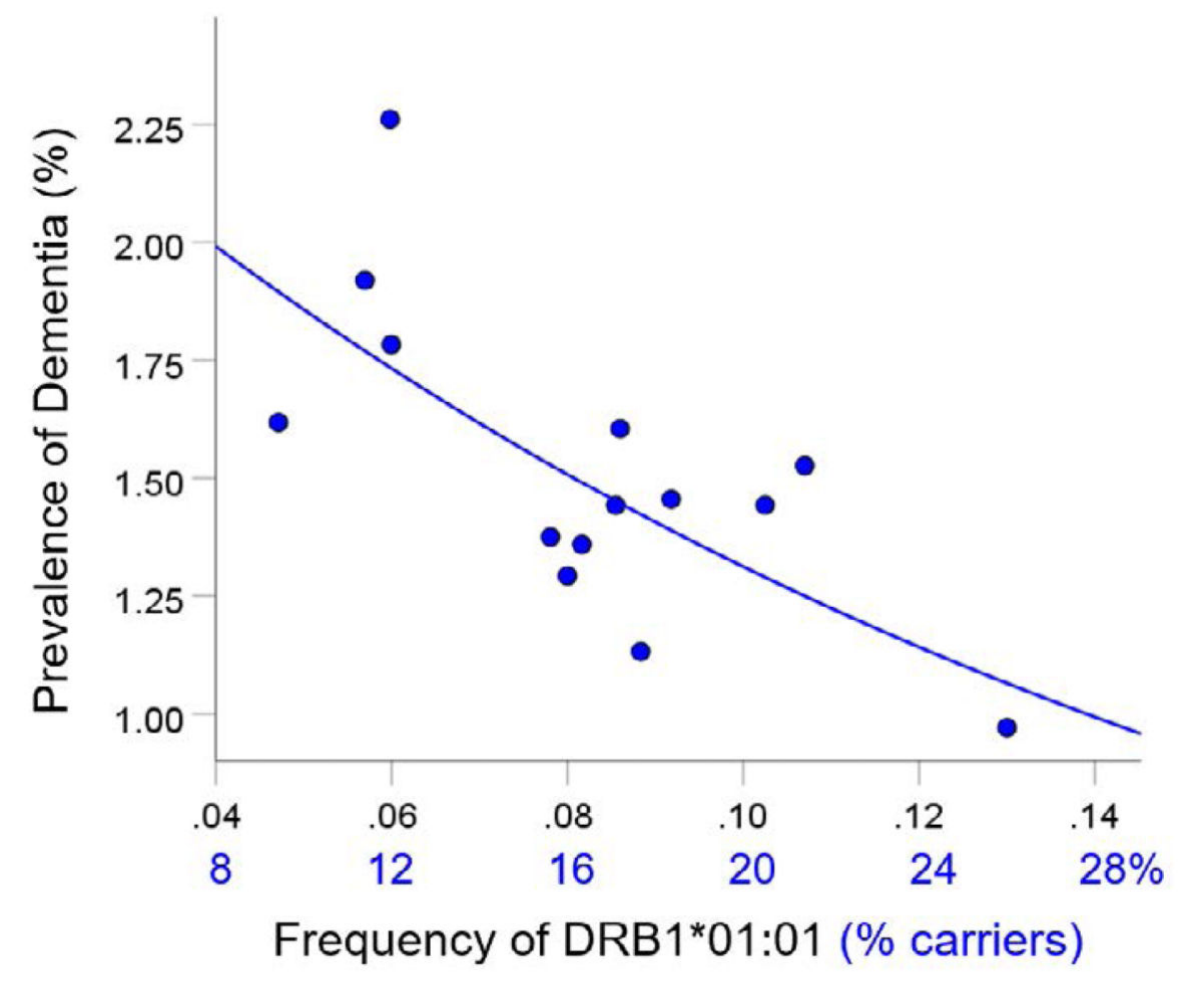
Dementia prevalence in 14 CWE countries is plotted against frequency of DRB1*01:01. See text for details.

**Figure 2.**
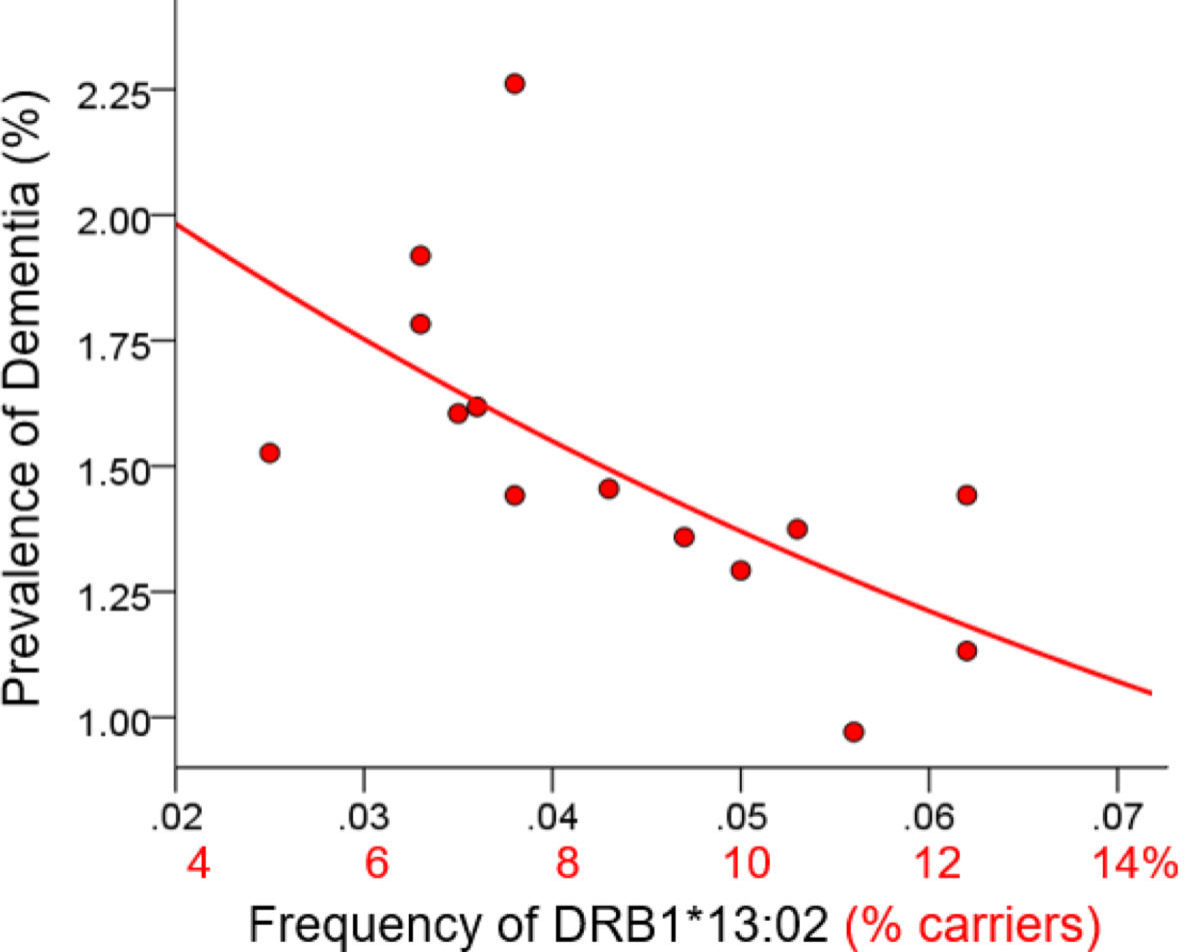
Dementia prevalence is plotted against frequency of DRB1*13:02. See text for details.

**Figure 3.**
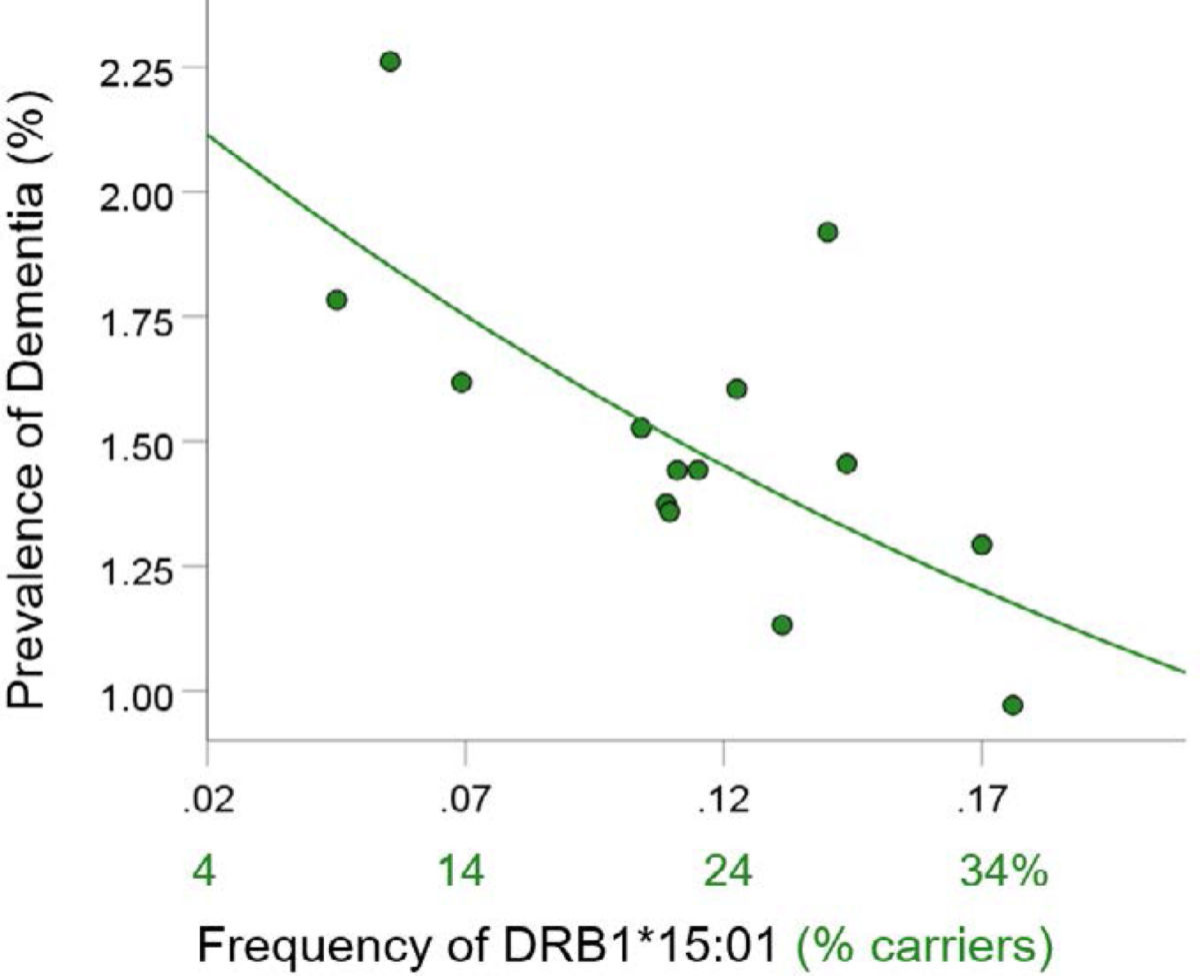
Dementia prevalence is plotted against frequency of DRB1*15:01. See text for details.

**Figure 4.**
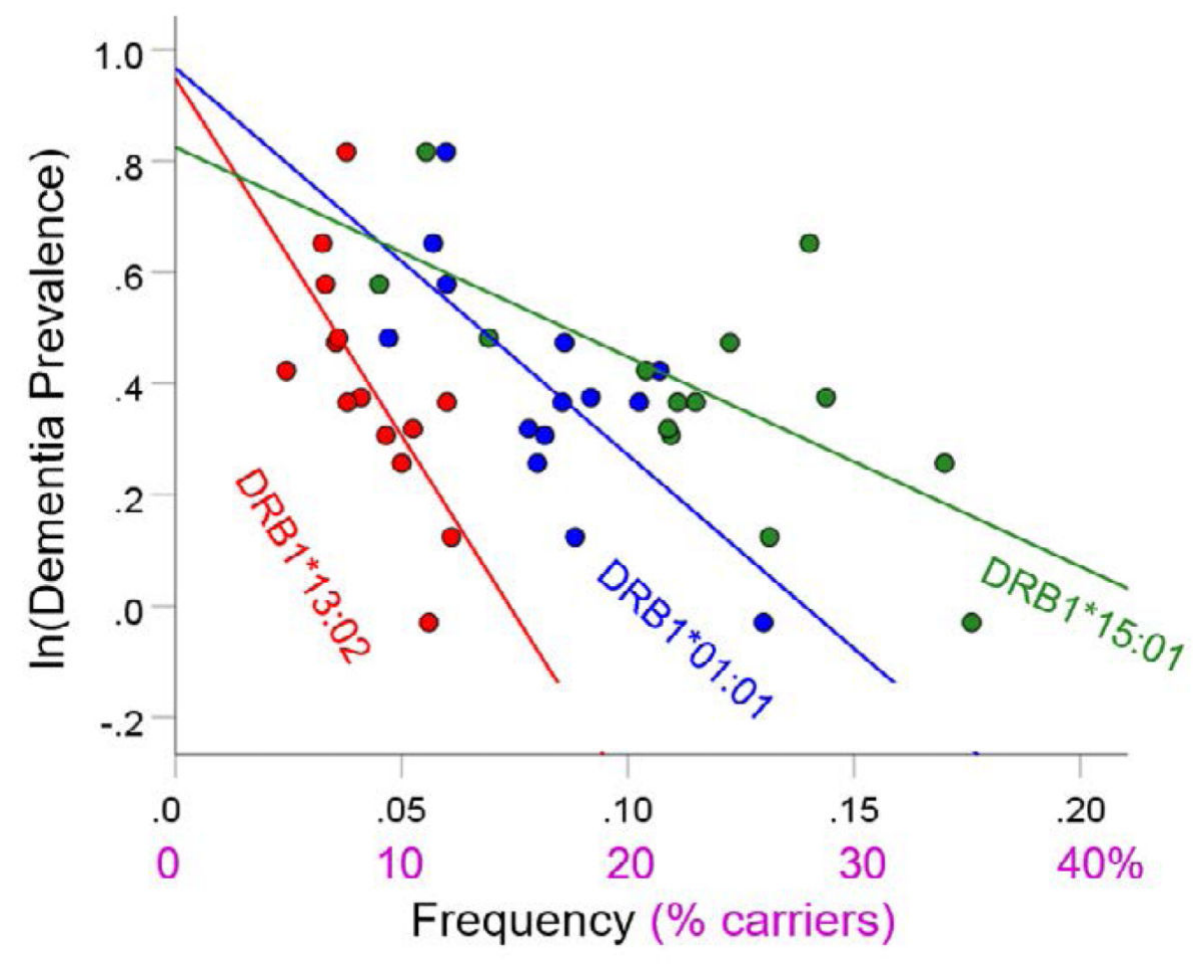
Illustration of the different effects of each allele on the natural log of dementia prevalence as evidenced by the varying steepness of the slopes.

For each of the three alleles, the prevalence of dementia decreased exponentially with increasing allele frequency (Figures. 1–3). In each case, the effects were highly statistically significant (DRB1*01:01: P = 0.003; DRB1*13:02: P = 0.008; DRB1*15:01: P = 0.007) and accounted for at least 46.8% of the variance in dementia prevalence (DRB1*01:01: ${\text{R}}^{2}=0.530$, Fig. 1; DRB1*13:02, ${\text{R}}^{2}=0.468$, Figure 2; DRB1*15:01: ${\text{R}}^{2}=0.471$, Figure 3). These effects were independent of apoE4 prevalence (partial correlation, with apoE4 prevalence partialed out: DRB1*01:01, r = −0.695, P = 0.008; DRB1*13:02, r = −0.782, P = 0.002; DRB1*15:01, r = −0.692, P = 0.016). Figure 4 illustrates differences in the magnitude of allele frequency effect on dementia prevalence as evidenced by the different slopes; of these, DRB1*13:02 had the steepest slope indicating that the protective effects for DRB1*13:02 are stronger than those for DRB1*01:01 and DRB1*15:01.

### Combined HLA effects on dementia prevalence

The frequencies of the three HLA alleles were uncorrelated (Spearman’s rho ranged from 0.38 to 0.46; P’s > 0.095); therefore, we summed the individual allele frequencies as an estimate of the population frequency for the presence of *any* of the three alleles (DRB1*01:01 *or* DRB1*13:02 *or* DRB1*15:01) and evaluated the effects of this sum on dementia prevalence:

(1) $$f_{\left[3\text{alleles}\right]}=f_{01:01}+f_{13:02}+f_{15:01}$$

(2) $$\text{Dementia Prevalence}(\text{\%})=2.92{\text{e}}^{-2.83f[3\text{alleles}]}$$

**Figure 5.**
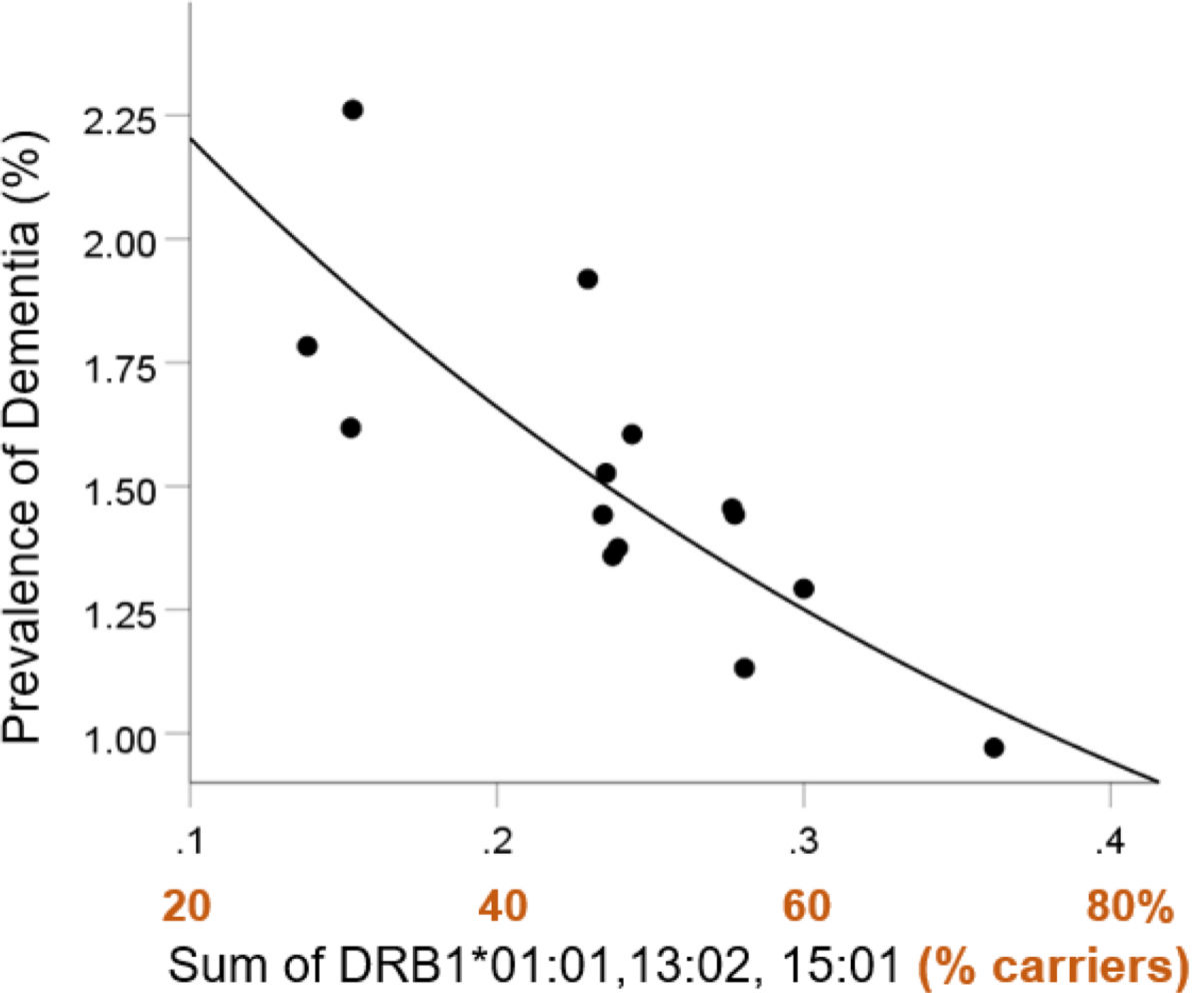
Dementia prevalence is plotted against the sum of the frequency of DRB1*01:01, DRB1*13:02, and DRB1*15:01. See text for details.

This model (Figure 5) was highly statistically significant (P=0.003) and explained 67.3% of the variance $({\text{R}}^{2}=0.673)$. By taking the natural logarithms (ln) on both sides, we get, equivalently,

(3) $$\ln(\text{Dementia Prevalence}\%)=\text{In}(2.92)-2.83f_{\left[3\text{alleles}\right]}=\;1.07-2.83f_{\left[3\text{alleles}\right]}$$

This effect was robust (Spearman’s rank correlation =−0.807, P=0.0005), even when the apoE4 frequency was partialed out (partial r=−0.806, P=0.00088).

**Figure 6.**
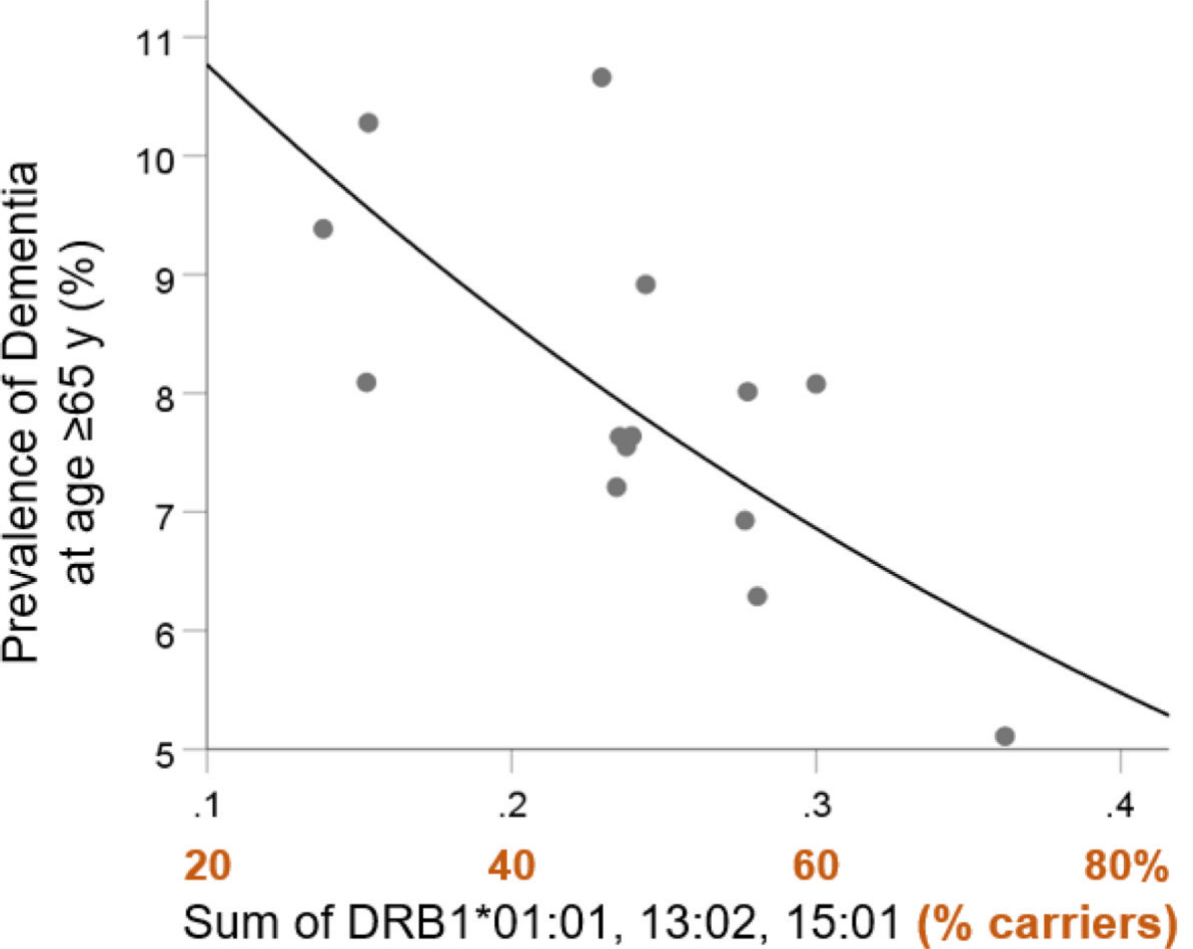
Dementia prevalence in those aged 65 and older is plotted against the sum of the frequency of DRB1*01:01, DRB1*13:02, and DRB1*15:01. See text for details.

We also evaluated the effects of the three alleles on dementia prevalence in those aged 65 and older. Similar to the above findings, the population frequency of the three alleles was significantly associated with dementia prevalence (Figure 6; P=0.004, ${\text{R}}^{2}=0.479$) and was highly protective:

(4) $$\text{Dementia Prevalence in}>65\text{y}(\text{\%})=13.48{\text{e}}^{-2.24f[3\text{alleles}]}$$

### HLA effects on change in dementia prevalence

**Figure 7.**
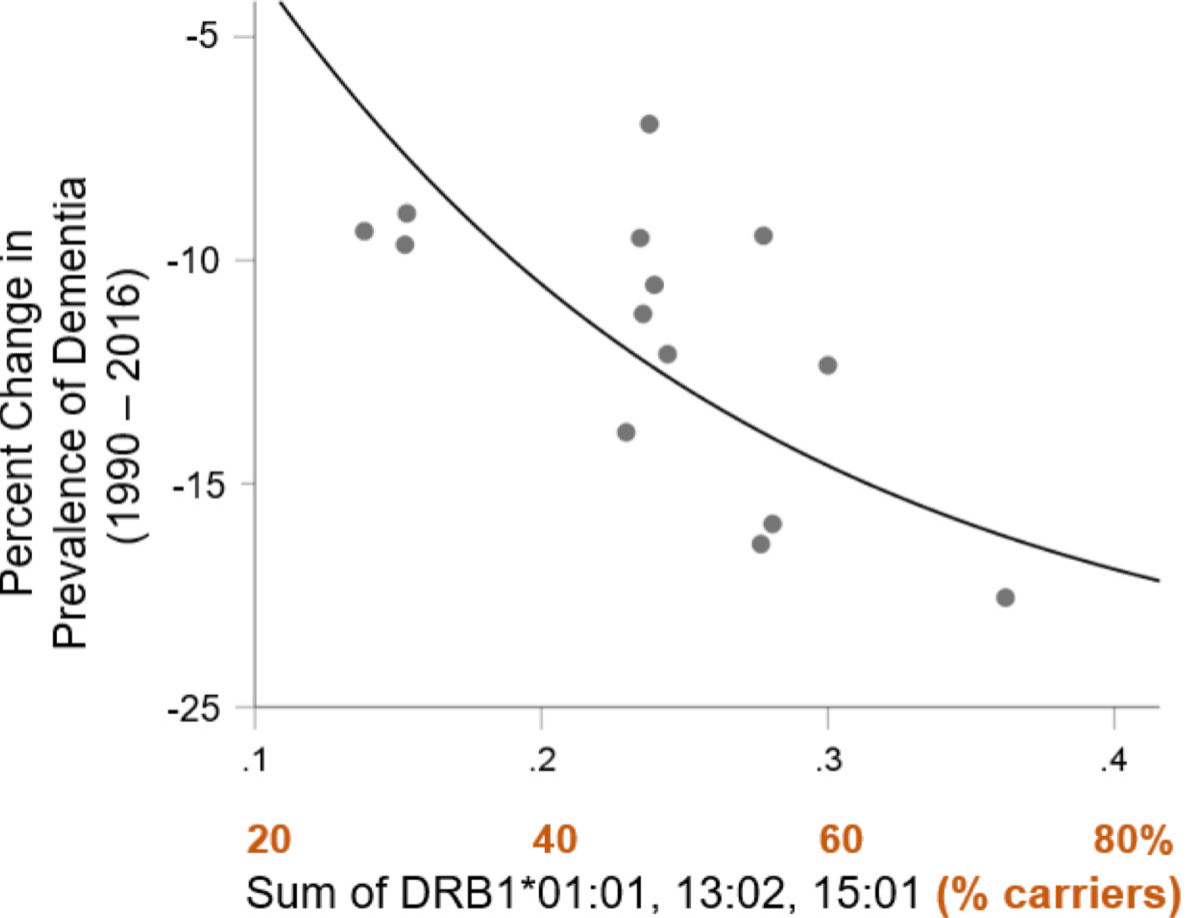
Change in dementia prevalence between 1990 and 2016 is plotted against the sum of the frequency of DRB1*01:01, DRB1*13:02, and DRB1*15:01. See text for details.

Finally, we evaluated the effects of the population frequency of these three alleles on the change in dementia prevalence between 1990 and 2016. Results of this analysis indicated that the sum of the population frequency of these three HLA alleles was significantly associated with a decrease in dementia prevalence observed over the last 26 years (P=0.006, ${\text{R}}^{2}=0.482$). That is, countries for which the frequency of these three alleles is higher saw a greater reduction in dementia prevalence during that time (Figure 7).

## Discussion

The results of the current study demonstrate robust protective effects of three common Class II (DRB1) HLA alleles on dementia prevalence in CWE. Since Class II HLA alleles play an essential role in antibody production and immunological memory to facilitate host protection in the event of re-exposure to viruses and other foreign pathogens, the effects observed here implicate foreign pathogens in dementia prevalence. That is, in countries for which the frequency of these three HLA alleles are relatively high, the reduced dementia prevalence is presumably due to enhanced pathogen elimination at the population level. Conversely, in countries for which the frequency of these three HLA alleles are relatively low, higher dementia prevalence may be partially due to pathogen persistence^3–6^. A number of viral and bacterial pathogens have been implicated in dementia^7,8^ though findings with regard to specific pathogens have often been inconsistent. In our lab, studies aimed at identifying antigens that bind to these three protective alleles and are highly immunogenic are currently underway. The findings of that line of study are expected to narrow the universe in terms of pathogens that may be causally related to dementia in the absence of immunogenetic protection provided by HLA DRB1*01:01, DRB1*13:02, and DRB1*15:01.

It is remarkable that 67% of the variance in dementia prevalence in CWE and 48% of the variance in the change in prevalence of dementia between 1990 and 2016 is accounted for by the three HLA alleles investigated here. It is likely that the remaining variance is attributable to other genetic influences and lifestyle factors such as diet and exercise. Indeed, modifiable risk factors are estimated to account for ~35% of dementia risk^25^. Thus, at the population level, these three HLA alleles coupled with lifestyle factors account for nearly all of the variance in dementia risk. Although apoE4 has been widely associated with dementia, prior studies have demonstrated that the effects of protective HLA alleles are independent of apoE4^1-3^. In light of that, we have speculated that apoE4 effects are secondary to HLA^4^. That is, we have proposed that given an HLA-antigen match (i.e., with sufficient binding affinity and immunogenicity) the risk associated with apoE4 status is minimized. However, in the absence of an HLA-antigen match, persistent antigens cause neural damage. Since one role of apoE is to facilitate neuronal repair^26,^ the damage caused by persistent antigens may stimulate neuronal repair. For apoE2 and apoE3 carriers, damaged neurons may be successfully repaired; however, given the neurotoxic effects of apoE4, additional neuronal damage may occur in apoE4 carriers. In this hypothesized model, the effects of apoE4 are downstream and HLA, instead, plays a crucial initial role.

## Conclusions

The current findings bolster prior work demonstrating immunogenetic protection against dementia and highlight identification and elimination of foreign antigens as a potential avenue for dementia prevention.

## Limitations

HLA is known to vary by region and ethnicity, presumably as a result of evolutionary adaptations related to population differences in pathogen exposure. Consequently, the highly protective effects of the three alleles studied here may not generalize worldwide. That is, it is likely that other protective HLA alleles will be identified in other regions or in populations not characterized primarily by European ancestry due to superior ability of those alleles in eliminating pathogens that are less common in CWE. Furthermore, the protective effects here are specific to dementia and do not preclude the possibility that these alleles are associated with risk for other conditions.

## Figures and Tables

**Table 1.** Dementia prevalence and life expectancy for Countries in Continental Western Europe.

Country	N Dementia^a^	N total population^b^	Fraction of population >65 y	N Population >65 y	Overall Dementia Prevalence	Estimated dementia prevalence in >65 y	Average life expectancy^b^
Austria	126914	8800000	0.18	1584000	1.442	8.012	81.5
Belgium	181350	11300000	0.18	2034000	1.605	8.916	81.5
Denmark	55336	5700000	0.19	1083000	0.971	5.109	80.5
Finland	83950	5500000	0.20	1100000	1.526	7.632	81.5
France	877760	64600000	0.18	11628000	1.359	7.549	82.0
Germany	1201668	82600000	0.21	17346000	1.455	6.928	80.5
Greece	192563	10800000	0.19	2052000	1.783	9.384	81.0
Italy	1370308	60600000	0.22	13332000	2.261	10.278	82.5
Netherlands	192425	17000000	0.18	3060000	1.132	6.288	81.5
Norway	67207	5200000	0.16	832000	1.292	8.078	82.0
Portugal	166660	10300000	0.20	2060000	1.618	8.090	80.0
Spain	830915	43300000	0.18	7794000	1.919	10.661	82.5
Sweden	142735	9900000	0.20	1980000	1.442	7.209	82.0
Switzerland	115476	8400000	0.18	1512000	1.375	7.637	83.0

a Data obtained from ref^21^.

b Data obtained from ref^22^.

**Table 2.** ApoE4 prevalence per country in continental Western Europe.^a^

Country	Sample size	ApoE4 prevalence
Austria	683	0.105
Belgium	1660	0.128
Denmark	1620	0.127
Finland	7285	0.215
France	8247	0.114
Germany	6123	0.132
Greece	216	0.065
Italy	4189	0.083
Netherlands	5484	0.154
Norway	1097	0.174
Portugal	381	0.169
Spain	4863	0.094
Sweden	686	0.205
Switzerland	2061	0.109

a .Data obtained from ref^23^.

**Table 3.** HLA allele frequencies per country in Continental Western Europe.^a^

Country	Sample size (2n)	DRB1*01:01 Frequency	DRB1*13:02 frequency	DRB1*15:01 frequency
Austria	400	0.102	0.062	0.115
Belgium	198	0.086	0.035	0.123 (2n=408)^b^
Denmark	110	0.130	0.056	0.176
Finland	482	0.107	0.025	0.104
France	1260	0.082	0.047	0.109
Germany	140300	0.092	0.043	0.144
Greece	1848	0.060	0.033	0.045
Italy	12562	0.060	0.038	0.055
Netherlands	3632	0.088	0.062	0.131
Norway	362	0.080	0.050	0.170
Portugal	550	0.047	0.036	0.069
Spain	3850	0.057	0.033	0.140
Sweden	568	0.085	0.038	0.111
Switzerland	41664	0.078	0.053	0.109

a . Obtained from allelefrequencies.net October 19, 2019.

b . Data from ref^24^.

## Data Availability

All data are available in references provided and at allelefrequencies.net.
